# NEOGRAN: traceable graph-text fusion for disease–protein relation prediction in biomedical knowledge graphs

**DOI:** 10.1186/s12859-026-06580-3

**Published:** 2026-08-08

**Authors:** Zhenxing Wang, Qihe Wang, Murong Zhou, Guohua Wang, Yuming Zhao

**Affiliations:** 1 College of Computer Science and Artificial Intelligence, Northeast Forestry University, Harbin, 150040 China; 2 Department of Computer Science and Technology, Harbin Institute of Technology, Harbin, 150001 China

**Keywords:** Biomedical knowledge graph, Disease–protein relation prediction, Graph–text fusion, Evidence path reasoning, Interpretable relation prediction

## Abstract

**Background:**

Accurately predicting disease–protein relations in biomedical knowledge graphs helps link disease phenotypes to molecular mechanisms and supports disease-related knowledge discovery and candidate target identification. Biomedical knowledge graphs organize multisource biomedical knowledge, including diseases, proteins, drugs, and pathways, as entity nodes and relational edges, providing a structured foundation for modeling complex biomedical associations. Existing methods are often constrained by single-modality modeling, shallow graph–text fusion, and insufficient traceable evidence, which limits their ability to exploit graph–text complementarity and weakens downstream validation and structural evidence interpretation.

**Results:**

To address these limitations, we propose NEOGRAN, a graph–text collaborative framework comprising three core modules for relation prediction in biomedical knowledge graphs. The dual-encoder architecture captures graph structural patterns and biomedical entity representations to mitigate single-modality modeling. The bidirectional cross-attention module enables deep graph–text interaction to overcome shallow fusion. The interpretable path module generates traceable evidence paths to support prediction verification, structural evidence interpretation, and hypothesis generation. On PrimeKG, NEOGRAN achieved an AUPR of 0.9860 and an AUROC of 0.9875 under the 1:1 sampled classification setting, and further obtained an MRR of 0.0735 in the all-candidate ranking evaluation. External validation on BioKG further shows that NEOGRAN remains effective under differences in entity coverage, relation composition, and local topology, supporting its method-level generalizability across knowledge graph sources.

**Conclusions:**

NEOGRAN provides an effective solution for relation prediction in biomedical knowledge graphs while offering traceable structural evidence for hypothesis generation and further biological validation.

## Introduction

Accurate prediction of latent relations in biomedical knowledge graphs can uncover missing biomedical associations, elucidate disease mechanisms, and prioritize candidate targets [1, 2]. With the continued growth of biomedical databases, clinical omics data, and literature-derived knowledge, biomedical knowledge graphs have become important for organizing multisource biomedical data into networked representations and discovering hidden associations [3]. By integrating diseases, genes/proteins, drugs, phenotypes, and their relations from literature, databases, and curated biomedical resources, biomedical knowledge graphs provide a structured foundation for modeling complex biomedical systems and data-driven drug discovery [4–6]. Relation prediction in biomedical knowledge graphs is therefore important for prioritizing hypotheses for mechanistic studies, candidate screening, and experimental validation [7, 8]. In disease-centered discovery, disease–protein relation prediction is particularly relevant because it links phenotypic abnormalities to molecular mechanisms while requiring both predictive plausibility and mechanistic verifiability [9, 10].

Several representative lines of work have advanced relation prediction in biomedical knowledge graphs. Knowledge graph embedding (KGE) methods, such as DistMult and ComplEx, learn low-dimensional representations of entities and relations for efficient link prediction, but they mainly rely on structural triples and scoring functions [11, 12]. Graph neural network (GNN)-based methods use neighborhood message passing to capture local topology and higher-order relational dependencies [13, 14], but they remain less effective in modeling node-associated text and external semantics [6, 8]. Biomedical pretrained language models, such as BioBERT and PubMedBERT, have improved biomedical text representation [15, 16] and promoted text-enhanced or multimodal relation prediction [17], but many methods still rely on weak graph–text coupling. The integration of large language models with medical knowledge graphs is also advancing medical semantic understanding, knowledge retrieval, and structured reasoning, but task-specific modeling is still needed to reduce knowledge noise and reasoning errors.

Despite these advances, robust relation prediction in biomedical knowledge graphs remains limited by three key challenges. First, disease–protein associations depend on both graph structural constraints [18, 19] and entity-level textual identifiers [20], making single-modality modeling insufficient for robust and verifiable prediction [21]. Second, existing graph–text fusion is often based on concatenation, score aggregation, or shallow interaction, which weakens the modeling of complementarity between structural neighborhoods and name/type-based textual cues [22, 23]. Third, high-confidence predictions rarely provide traceable evidence paths aligned with the prediction process, limiting prediction verification, interpretable relation reasoning [24, 25], structural evidence interpretation, and downstream validation planning [10]. A suitable solution should therefore combine structural–textual representation learning, deep graph–text interaction, and traceable evidence generation within a unified relation prediction framework.

To address these challenges, we propose NEOGRAN, a graph–text fusion framework for relation prediction in biomedical knowledge graphs. NEOGRAN jointly uses structurally derived relational information and textual cues through three core modules: a dual-encoder architecture, a bidirectional cross-attention module, and an interpretable path module. The dual-encoder architecture learns graph-based structural representations and name/type-based textual representations. Specifically, the structural encoder captures relation-aware neighborhood patterns, while the text encoder encodes entity names and entity types as lightweight biomedical textual identifiers. The bidirectional cross-attention module aligns the two representation spaces through mutual Structural-to-Textual and Textual-to-Structural attention, enabling deeper graph–text complementarity beyond shallow fusion. The resulting unified node representations are used for relation scoring and candidate disease–protein association prediction. The interpretable path module traces evidence paths during relation prediction and connects predicted node pairs with supporting graph contexts, improving prediction verification, structural evidence interpretation, and downstream validation planning.

Experimental results on PrimeKG show that NEOGRAN achieves strong sampled-classification performance and favorable early-ranking ability compared with representative graph-based, text-enhanced, and multimodal methods. External validation on BioKG further shows that NEOGRAN remains effective under differences in entity coverage, relation composition, and local topology, supporting its method-level generalizability across knowledge graph sources. The main contributions of this paper are summarized as follows:

- We propose NEOGRAN, a graph–text fusion framework for relation prediction in biomedical knowledge graphs. It uses a dual-encoder architecture to jointly model graph structural information and entity-level textual representations, thereby mitigating the limitations of single-modality modeling.
- We propose a bidirectional cross-attention module for deep graph–text interaction. This module aligns structural and textual representations in both directions and strengthens cross-modal complementarity beyond shallow fusion.
- We propose an interpretable path module for traceable evidence generation. This module enables NEOGRAN to output evidence paths for predicted node pairs, supporting prediction verification, structural evidence interpretation, and hypothesis generation.

## Related work

Disease–protein relation prediction in biomedical knowledge graphs is a translational bioinformatics task that links phenotype-level disease knowledge with molecular mechanisms and candidate targets. Unlike general knowledge graph completion, this task involves heterogeneous biomedical entities, typed relations, sparse target links, incomplete annotations, and uneven entity descriptions [26]. Structural-only prediction may ignore mechanistic clues in entity text, whereas text-only prediction may fail to exploit explicit relational constraints. Therefore, effective models need to jointly use graph structure, biomedical semantics, and traceable evidence under carefully defined evaluation splits.

### Single-encoder modeling methods

Single-encoder structural methods provide a basic solution for biomedical relation prediction. Knowledge graph embedding methods, such as DistMult and ComplEx, represent entities and relations in low-dimensional spaces and score candidate triples, but they mainly depend on structural triples and have limited ability to model heterogeneous neighborhoods or biomedical semantics [11, 12]. Graph neural network methods improve structural modeling through message passing and can capture local topology, higher-order dependencies, and relation-specific edge types [13]. Recent studies further extend structural modeling to subgraph-focused embedding and community-level abstraction, showing that biomedical relation prediction can benefit from richer local graph contexts [27, 28]. Nevertheless, structure-centered encoders remain limited when node-associated descriptions, functional annotations, or disease definitions provide important semantic evidence.

Semantic modeling methods complement structural encoders by using biomedical entity descriptions and literature-derived context. Biomedical pretrained language models such as BioBERT and PubMedBERT provide domain-specific text representations for biomedical concepts, disease terms, and protein functions [15, 16]. Medical knowledge graph completion has incorporated entity descriptions and type information for semantic and type-aware relation modeling [19], while BioBridge connects biomedical foundation models through knowledge graphs across protein, molecular, and clinical representation spaces [29]. Medical retrieval-augmented generation and graph-based reasoning further show the value of combining language models with structured biomedical knowledge [30, 31]. Structure-aware tuning also indicates that language models can benefit from explicit graph constraints [32, 33]. However, semantic encoders or LLM-based retrieval alone cannot replace graph relational constraints or ensure that predicted disease–protein relations are supported by traceable structural evidence.

### Multimodal fusion methods

Multimodal fusion methods aim to improve knowledge graph relation prediction by integrating structural representations with textual or auxiliary modality information. General multimodal knowledge graph completion has evolved from simple feature concatenation to interaction-aware, relation-aware, expert-based, and adaptive fusion strategies, as represented by MCCI, MESN, and related dynamic fusion methods [23, 34–36]. However, many of these methods are designed for open-domain knowledge graphs or image–text–structure settings, where the modality assumptions differ from biomedical graph–text relation prediction. In biomedical knowledge graphs, node-associated text is often sparse and identifier-like, and its utility depends on how it is coordinated with typed relational neighborhoods during candidate-pair scoring.

Recent biomedical graph and knowledge graph methods provide important foundations for disease-related relation prediction. FuseLinker [37] introduces language-model-derived textual embeddings into GNN-based link prediction and demonstrates the value of incorporating textual representations into biomedical graph modeling. BioMedKG [38] combines language-model representations, graph contrastive learning, and knowledge graph embeddings to support multimodal biomedical representation learning. BioGraphFusion [20] coordinates graph and semantic representations for biomedical completion and reasoning. TxGNN [6] is a strong biomedical graph neural network model that focuses on graph-based relation prediction in biomedical knowledge graphs. KDGene [8] is a representative disease–gene prediction model based on knowledge graph representation learning, with a particular focus on predicting disease–gene/protein associations in biomedical knowledge graphs. Together, these studies show that structural modeling, textual representation, and biomedical knowledge graph learning are all useful for relation prediction. They also motivate a more explicit investigation of how relation-aware structural neighborhoods and entity-level name/type textual cues can be mutually conditioned within the same disease–protein candidate-pair scoring process.

Path-oriented reasoning methods and GraphRAG-style retrieval can further provide graph-structured evidence for biomedical prediction tasks [39–41]. Nevertheless, retrieved or inferred paths are often separated from the representation learning process used for relation scoring. Building on these advances, NEOGRAN integrates relation-aware structural encoding, name/type-based textual encoding, bidirectional cross-modal conditioning between sampled structural memory and token-level textual representations, gated node-pair fusion, and native evidence-path tracing within a unified disease–protein relation prediction framework.

## Methods

### Problem formulation

Disease–protein association prediction on biomedical knowledge graphs is formalized as a supervised graph–text relation prediction problem with traceable evidence generation. Let the biomedical knowledge graph be denoted by ${\mathcal {G}}=({\mathcal {V}},{\mathcal {E}},{\mathcal {R}})$, where ${\mathcal {V}}$ is the set of entity nodes, ${\mathcal {R}}$ is the set of relation types, and ${\mathcal {E}}\subseteq {\mathcal {V}}\times {\mathcal {R}}\times {\mathcal {V}}$ is the set of typed directed edges. This study focuses on a target disease–protein relation $r^{*}$. For a candidate node pair (*i*, *j*), where *i* is a disease node and *j* is a protein node, each node has both a structural neighborhood and node-associated text, denoted by $\textrm{text}(i)$ and $\textrm{text}(j)$. The node-associated text is constructed from entity names and entity types. The goal is to learn a prediction function for $r^{*}$ under incomplete graph structure, sparse relational links, and heterogeneous node semantics. Given the graph structure and node-associated texts, the model outputs a probability $p_{ij}\in [0,1]$ indicating the confidence that $r^{*}$ holds for (*i*, *j*), with a binary label $y_{ij}\in \{0,1\}$ in supervised learning. Unlike structural-only link prediction, this study further requires the model to output a traceable evidence path $\pi _{ij}^{*}$ for each node pair to support subsequent validation and structural evidence analysis. The main notation used in the methodology section is summarized in Table 1.

**Table 1:** Notation summary for the methodology section

Symbol	Description
${\mathcal {G}}$	Biomedical knowledge graph
${\mathcal {V}}$	Set of entity nodes
${\mathcal {E}}$	Set of typed directed edges
${\mathcal {R}}$	Set of relation types
$r^{*}$	Target disease–protein relation
(*i*, *j*)	Candidate disease–protein node pair
$\textrm{text}(i)$	Node-associated text of node *i* constructed from entity name and type
$y_{ij}$	Binary label for candidate node pair (*i*, *j*)
$p_{ij}$	Predicted probability that $r^{*}$ holds for (*i*, *j*)
${\mathbf{h}}_{i}^{(l)}$	Structural representation of node *i* at R-GCN layer *l*
${\mathbf{z}}_{i}^{g}$	Projected structural representation of node *i*
${\mathbf{H}}_{i}$	Token-level textual representation matrix of node *i*
${\mathbf{z}}_{i}^{t}$	Projected textual representation of node *i*
${\mathbf{C}}_{i}$	Token-level textual context used in Structural-to-Textual attention
${\mathbf{G}}_{i}$	Sampled local structural memory used in Textual-to-Structural attention
$\bar{{\mathbf{z}}}_{i}^{g}$	Textual-cue-enhanced structural representation of node *i*
$\bar{{\mathbf{z}}}_{i}^{t}$	Structure-enhanced textual representation of node *i*
$\boldsymbol{\alpha }_{i}$	Dimension-wise gating vector for fusing structural and textual representations
${\mathbf{e}}_{i}$	Final fused representation of node *i*
$\pi _{ij}^{*}$	Traceable evidence path for candidate node pair (*i*, *j*)

### Overview of NEOGRAN

**Fig. 1:**
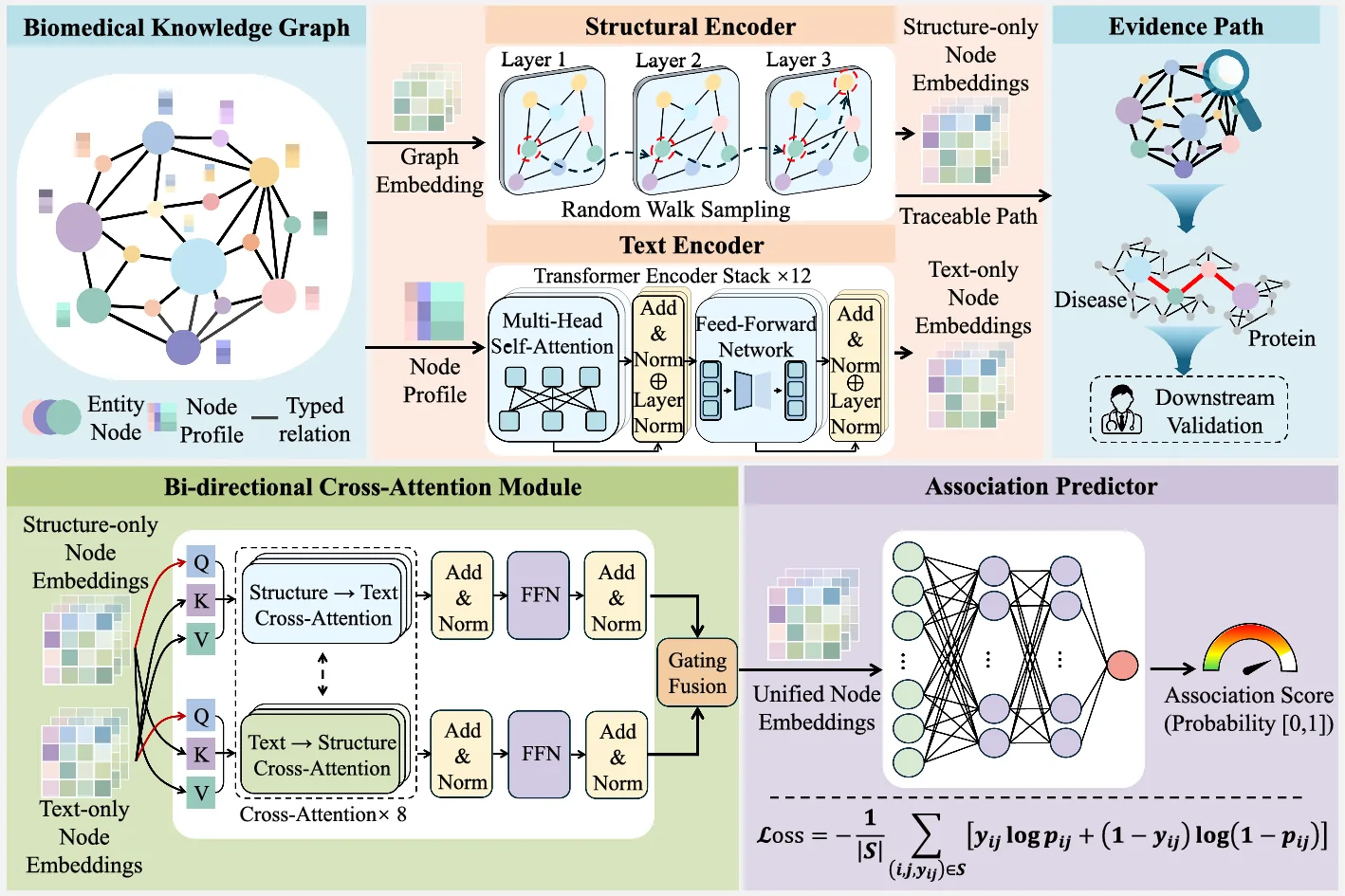
Overall framework of NEOGRAN. The model takes a biomedical knowledge graph and entity-level node texts as input. The structural branch uses a relation-aware structural encoder to learn node representations informed by graph topology and relation types, whereas the textual branch uses a biomedical text encoder to encode entity-level textual cues derived from node names and types. The bidirectional cross-attention module contains Structural-to-Textual attention, which conditions structural representations on entity-level textual cues, and Textual-to-Structural attention, which conditions textual representations on sampled local structural memory. The gated fusion module integrates the two updated representations into unified node representations for candidate disease–protein pair scoring. The relation predictor outputs the probability of a disease–protein relation, while the evidence path tracing module provides traceable structural paths associated with the prediction

NEOGRAN is a unified graph–text collaborative framework for disease–protein relation prediction with traceable evidence support. As shown in Fig. 1, NEOGRAN takes ${\mathcal {G}}$ and node-associated texts $\textrm{text}(i)$ and $\textrm{text}(j)$ as inputs, predicts whether a candidate disease–protein node pair (*i*, *j*) holds $r^{*}$, and outputs both the relation probability $p_{ij}$ and an evidence path $\pi _{ij}^{*}$. The framework contains three modules. First, the dual-encoder architecture learns structural representations from heterogeneous graph neighborhoods and textual representations from entity names and entity types. Second, the bidirectional cross-attention module aligns and refines the two representation spaces in both directions, allowing graph neighborhoods to select relevant textual cues and textual contexts to incorporate relational constraints. The resulting unified node representations are used for relation scoring under $r^{*}$. Third, the interpretable path module traces evidence paths from structural propagation and connects the candidate node pair through relevant intermediate entities and relations, providing support for literature-based review, hypothesis generation, and further biological validation.

### Dual-encoder architecture

Disease–protein relations are shaped by multi-hop relational structures and entity-level textual identifiers such as entity names and types. Graph structure provides relational dependencies and neighborhood context, whereas textual information provides complementary entity-level cues but lacks explicit topological constraints. Because structural-only or text-only encoders cannot capture both information sources, NEOGRAN uses a dual-encoder architecture to learn structural and textual node representations for later cross-modal interaction and fusion.

At the input stage, each node profile is constructed by concatenating the entity name and entity type. This profile generates the node-associated text $\textrm{text}(i)$. In implementation, $\textrm{text}(i)=\operatorname {Concat}(\textrm{name}_i,\textrm{type}_i)$. Disease definitions, protein functional descriptions, literature snippets, and relation-specific textual descriptions are not used as model input. This design provides lightweight entity-level textual identifiers while avoiding the direct use of explicit disease–protein relation descriptions in node-associated text.

Structurally, the dual encoder contains two parallel branches that map different input spaces into the same *D*-dimensional representation space. The structural branch can be written as

1 $$\begin{aligned} f_g({\mathcal {G}}, i) = {\mathbf{z}}_{i}^{g} \in {\mathbb {R}}^{D}, \end{aligned}$$

where $f_g(\cdot )$ denotes the relation-aware graph encoder defined by the R-GCN propagation and graph projection steps below. The textual branch can be written as

2 $$\begin{aligned} f_t(\textrm{text}(i)) = ({\mathbf{H}}_{i},{\mathbf{z}}_{i}^{t}), \quad {\mathbf{z}}_{i}^{t}\in {\mathbb {R}}^{D}, \end{aligned}$$

where ${\mathbf{H}}_{i}$ is the token-level PubMedBERT representation matrix and ${\mathbf{z}}_{i}^{t}$ is the pooled and projected textual node representation. The two branches are first encoded independently and then coupled by bidirectional cross-attention.

#### Structure encoder

The structural encoder learns relation-aware node representations from ${\mathcal {G}}=({\mathcal {V}},{\mathcal {E}},{\mathcal {R}})$ using a multi-layer relational graph convolutional network (R-GCN). Let the representation of node *i* at layer *l* be ${\mathbf{h}}_{i}^{(l)}\in {\mathbb {R}}^{d_l}$, where $d_l$ is the feature dimensionality and ${\mathbf{h}}_{i}^{(0)}$ is the initial node representation. If continuous attribute features ${\mathbf{x}}_i$ are available, they are combined with the node type embedding. Otherwise, a learnable entity embedding is combined with the type embedding:

3 $$\begin{aligned} {\mathbf{h}}_{i}^{(0)} = {\left\{ \begin{array}{ll} {\mathbf{P}}_{x}\left[ {\mathbf{x}}_{i}\Vert {\mathbf{u}}_{i}^{\textrm{type}}\right] , & \text {if continuous attributes are available},\\ {\mathbf{P}}_{e}\left[ {\mathbf{u}}_{i}^{\textrm{id}}\Vert {\mathbf{u}}_{i}^{\textrm{type}}\right] , & \text {otherwise} \end{array}\right. }, \end{aligned}$$

where ${\mathbf{u}}_{i}^{\textrm{id}}\in {\mathbb {R}}^{d_{\textrm{id}}}$ and ${\mathbf{u}}_{i}^{\textrm{type}}\in {\mathbb {R}}^{d_{\textrm{type}}}$ denote the learnable entity embedding and node type embedding, respectively. The projection matrices ${\mathbf{P}}_{x}\in {\mathbb {R}}^{d_0\times (d_x+d_{\textrm{type}})}$ and ${\mathbf{P}}_{e}\in {\mathbb {R}}^{d_0\times (d_{\textrm{id}}+d_{\textrm{type}})}$ map the two initialization inputs into the same $d_0$-dimensional structural space, and $[\cdot \Vert \cdot ]$ denotes vector concatenation.

For any relation type $r\in {\mathcal {R}}$, let the incoming neighbors of node *i* be ${\mathcal {N}}_{i}^{r}=\{j\mid (j,r,i)\in {\mathcal {E}}\}$. At layer *l*, relation-specific transformations are applied to neighbor representations and aggregated at node *i*:

4 $$\begin{aligned} \tilde{{\mathbf{h}}}_{i}^{(l+1)} = {\mathbf{W}}_{0}^{(l)}{\mathbf{h}}_{i}^{(l)} + \sum _{r\in {\mathcal {R}}} \sum _{j\in {\mathcal {N}}_{i}^{r}} \frac{1}{c_{i,r}} {\mathbf{W}}_{r}^{(l)}{\mathbf{h}}_{j}^{(l)}, \end{aligned}$$

where ${\mathbf{W}}_{0}^{(l)}\in {\mathbb {R}}^{d_{l+1}\times d_l}$ is the self-loop transformation matrix, ${\mathbf{W}}_{r}^{(l)}\in {\mathbb {R}}^{d_{l+1}\times d_l}$ is the transformation matrix for relation *r*, and $c_{i,r}=\max (1,|{\mathcal {N}}_{i}^{r}|)$ is the normalization coefficient.

Layer normalization, nonlinear activation, and dropout are then applied:

5 $$\begin{aligned} {\mathbf{h}}_{i}^{(l+1)} = \operatorname {Dropout} \left( \operatorname {ReLU} \left( \operatorname {LN} \left( \tilde{{\mathbf{h}}}_{i}^{(l+1)} \right) \right) \right) , \end{aligned}$$

where $\operatorname {LN}(\cdot )$, $\operatorname {ReLU}(\cdot )$, and $\operatorname {Dropout}(\cdot )$ denote Layer Normalization, the nonlinear activation function, and the dropout operation, respectively.

After *L* relation propagation layers, the final structural representation ${\mathbf{h}}_{i}^{(L)}$ is mapped into the unified representation space ${\mathbb {R}}^{D}$:

6 $$\begin{aligned} {\mathbf{z}}_{i}^{g} = {\mathbf{P}}_{g}{\mathbf{h}}_{i}^{(L)}, \end{aligned}$$

where ${\mathbf{P}}_{g}\in {\mathbb {R}}^{D\times d_L}$ is a learnable projection matrix, $d_L$ is the output dimension of layer *L*, and ${\mathbf{z}}_{i}^{g}\in {\mathbb {R}}^{D}$ is the structural representation of node *i*.

To align with Textual-to-Structural cross-attention, a structural context is constructed for each node. Because full neighborhoods in large biomedical knowledge graphs are computationally costly and may be dominated by high-degree nodes or frequent relations, random walk sampling is performed on the message-passing graph. Specifically, $N_w$ random walks of length $\ell _w$ start from node *i*, and the *m*-th walk is

7 $$\begin{aligned} w_i^{(m)} = \left( i=v_{0}^{(m)}, r_{1}^{(m)}, v_{1}^{(m)}, \ldots , r_{\ell _w}^{(m)}, v_{\ell _w}^{(m)} \right) . \end{aligned}$$

Visited nodes are collected and filtered by visit frequency, hop distance, and relation type coverage. At most $M_i$ nodes are retained as the structural context set:

8 $$\begin{aligned} {\mathcal {C}}_{i}^{g} = \{k_1,k_2,\ldots ,k_{M_i}\}. \end{aligned}$$

If fewer than $M_i$ nodes are sampled, all available nodes are retained. Random walks are performed only on the message-passing graph used for structural propagation, and target test edges are not used as target-relation edges in structural context sampling.

#### Text encoder

Graph structure alone cannot capture entity-level textual identifiers such as biomedical names and node types. Therefore, NEOGRAN uses a biomedical text encoder to encode the name- and type-based node profiles and produce textual representations that complement structural representations. For any node *i*, $\textrm{text}(i)$ is fed into PubMedBERT to obtain the token-level contextual representation matrix ${\mathbf{H}}_{i}\in {\mathbb {R}}^{T_i\times D_t}$, where $T_i$ is the text sequence length, $D_t$ is the hidden dimension of PubMedBERT, and ${\mathbf{H}}_{i}[t]\in {\mathbb {R}}^{D_t}$ is the contextual representation of the *t*-th token. Average pooling summarizes token-level representations, and the pooled representation is mapped into the unified representation space:

9 $$\begin{aligned} {\mathbf{z}}_{i}^{t} = {\mathbf{P}}_{t} \left( \frac{1}{T_i} \sum _{t=1}^{T_i} {\mathbf{H}}_{i}[t] \right) , \end{aligned}$$

where ${\mathbf{P}}_{t}\in {\mathbb {R}}^{D\times D_t}$ is a learnable projection matrix, *D* is consistent with Eq. (6), and ${\mathbf{z}}_{i}^{t}\in {\mathbb {R}}^{D}$ denotes the textual representation of node *i*. The structural representation ${\mathbf{z}}_{i}^{g}$ and textual representation ${\mathbf{z}}_{i}^{t}$ are then aligned and fused for relation prediction.

### Bidirectional cross-modal interaction and gated fusion

The dual-encoder architecture produces structural and textual representations from different information spaces. Structural information can benefit from entity-level textual cues, while textual representations require graph constraints to assess relevance to candidate relations. Because simple concatenation, weighted averaging, and one-way attention cannot fully capture this bidirectional dependency, NEOGRAN uses Structural-to-Textual and Textual-to-Structural cross-attention followed by gated fusion.

At this stage, NEOGRAN has obtained ${\mathbf{z}}_{i}^{g}\in {\mathbb {R}}^{D}$ and ${\mathbf{z}}_{i}^{t}\in {\mathbb {R}}^{D}$. The token-level matrix ${\mathbf{H}}_{i}\in {\mathbb {R}}^{T_i\times D_t}$ is first projected into the unified space:

10 $$\begin{aligned} {\mathbf{C}}_{i} = {\mathbf{H}}_{i}{\mathbf{W}}_{c}, \end{aligned}$$

where ${\mathbf{W}}_{c}\in {\mathbb {R}}^{D_t\times D}$ is a learnable projection matrix and ${\mathbf{C}}_{i}\in {\mathbb {R}}^{T_i\times D}$ is the textual context representation.

Given ${\mathcal {C}}_{i}^{g}=\{k_1,k_2,\ldots ,k_{M_i}\}$, the corresponding structural representations are stacked as

11 $$\begin{aligned} {\mathbf{G}}_{i} = \left[ {\mathbf{z}}_{k_1}^{g}, {\mathbf{z}}_{k_2}^{g}, \ldots , {\mathbf{z}}_{k_{M_i}}^{g} \right] ^{\top }, \end{aligned}$$

where ${\mathbf{G}}_{i}\in {\mathbb {R}}^{M_i\times D}$ provides local structural memory for Textual-to-Structural cross-attention.

The multi-head attention operator used in both directions is implemented as scaled dot-product attention. For a query matrix ${\mathbf{Q}}$, a key matrix ${\mathbf{K}}$, and a value matrix ${\mathbf{V}}$, the *a*-th attention head is computed as

12 $$\begin{aligned} \operatorname {head}_{a} = \operatorname {softmax} \left( \frac{ ({\mathbf{Q}}{\mathbf{W}}_{a}^{Q}) ({\mathbf{K}}{\mathbf{W}}_{a}^{K})^{\top } }{\sqrt{d_h}} \right) {\mathbf{V}}{\mathbf{W}}_{a}^{V}, \end{aligned}$$

where $d_h$ is the dimensionality of each attention head. The outputs of all heads are concatenated and projected as

13 $$\begin{aligned} \operatorname {MHA}({\mathbf{Q}},{\mathbf{K}},{\mathbf{V}}) = \operatorname {Concat} ( \operatorname {head}_{1}, \ldots , \operatorname {head}_{A} ) {\mathbf{W}}^{O}. \end{aligned}$$

In the Structural-to-Textual direction, the query, key, and value are set as ${\mathbf{Q}}={\mathbf{z}}_{i}^{g}$ and ${\mathbf{K}}={\mathbf{V}}={\mathbf{C}}_{i}$. In the Textual-to-Structural direction, they are set as ${\mathbf{Q}}={\mathbf{z}}_{i}^{t}$ and ${\mathbf{K}}={\mathbf{V}}={\mathbf{G}}_{i}$. Here, ${\mathbf{z}}_{i}^{g}$ and ${\mathbf{z}}_{i}^{t}$ are treated as length-one query sequences in the attention computation. Thus, the two directions implement different conditioning operations: the former updates the structural representation using textual context, whereas the latter updates the textual representation using sampled local structural memory.

In the Structural-to-Textual direction, ${\mathbf{z}}_{i}^{g}$ is used as the query, while ${\mathbf{C}}_{i}$ is used as both key and value. The single-vector query is treated as a length-one sequence, and the textual-cue-enhanced structural representation is computed as

14 $$\begin{aligned} \hat{{\mathbf{z}}}_{i}^{g} = \operatorname {LN} \left( {\mathbf{z}}_{i}^{g} + \operatorname {MHA} \left( {\mathbf{z}}_{i}^{g}, {\mathbf{C}}_{i}, {\mathbf{C}}_{i} \right) \right) , \end{aligned}$$

15 $$\begin{aligned} \bar{{\mathbf{z}}}_{i}^{g} = \operatorname {LN} \left( \hat{{\mathbf{z}}}_{i}^{g} + \operatorname {FFN} \left( \hat{{\mathbf{z}}}_{i}^{g} \right) \right) , \end{aligned}$$

where $\operatorname {MHA}(\cdot )$ denotes multi-head attention with inputs ordered as query, key, and value, $\operatorname {LN}(\cdot )$ denotes Layer Normalization, $\operatorname {FFN}(\cdot )$ denotes the feed-forward network, and $\bar{{\mathbf{z}}}_{i}^{g}\in {\mathbb {R}}^{D}$ denotes the final textual-cue-enhanced structural representation.

In the Textual-to-Structural direction, ${\mathbf{z}}_{i}^{t}$ is used as the query, while ${\mathbf{G}}_{i}$ is used as both key and value. The structure-enhanced textual representation is computed as

16 $$\begin{aligned} \hat{{\mathbf{z}}}_{i}^{t} = \operatorname {LN} \left( {\mathbf{z}}_{i}^{t} + \operatorname {MHA} \left( {\mathbf{z}}_{i}^{t}, {\mathbf{G}}_{i}, {\mathbf{G}}_{i} \right) \right) , \end{aligned}$$

17 $$\begin{aligned} \bar{{\mathbf{z}}}_{i}^{t} = \operatorname {LN} \left( \hat{{\mathbf{z}}}_{i}^{t} + \operatorname {FFN} \left( \hat{{\mathbf{z}}}_{i}^{t} \right) \right) , \end{aligned}$$

where $\hat{{\mathbf{z}}}_{i}^{t}\in {\mathbb {R}}^{D}$ is the intermediate textual representation updated with structural context and $\bar{{\mathbf{z}}}_{i}^{t}\in {\mathbb {R}}^{D}$ is the final structure-enhanced textual representation. Thus, structural representations interact with token-level textual cues, while textual representations incorporate local relational constraints from random-walk structural context.

After obtaining $\bar{{\mathbf{z}}}_{i}^{g}$ and $\bar{{\mathbf{z}}}_{i}^{t}$, a feature-level gating vector is computed:

18 $$\begin{aligned} \boldsymbol{\alpha }_{i} = \sigma \left( {\mathbf{W}}_{f} \left[ \bar{{\mathbf{z}}}_{i}^{g} \Vert \bar{{\mathbf{z}}}_{i}^{t} \right] + {\mathbf{b}}_{f} \right) , \end{aligned}$$

where $[\cdot \Vert \cdot ]$ denotes vector concatenation, ${\mathbf{W}}_{f}\in {\mathbb {R}}^{D\times 2D}$ and ${\mathbf{b}}_{f}\in {\mathbb {R}}^{D}$ are the weight matrix and bias vector, $\sigma (\cdot )$ denotes the Sigmoid function, and $\boldsymbol{\alpha }_{i}\in (0,1)^{D}$ is a dimension-wise gating vector. The unified node representation is

19 $$\begin{aligned} {\mathbf{e}}_{i} = \boldsymbol{\alpha }_{i}\odot \bar{{\mathbf{z}}}_{i}^{g} + \left( {\mathbf{1}}-\boldsymbol{\alpha }_{i} \right) \odot \bar{{\mathbf{z}}}_{i}^{t}, \end{aligned}$$

where *⊙ * denotes element-wise multiplication, ${\mathbf{1}}\in {\mathbb {R}}^{D}$ denotes the all-one vector, and ${\mathbf{e}}_{i}\in {\mathbb {R}}^{D}$ integrates graph structure and textual information.

### Relation prediction with native path tracing

After fusion, NEOGRAN obtains unified node representations ${\mathbf{e}}_{i}\in {\mathbb {R}}^{D}$ and ${\mathbf{e}}_{j}\in {\mathbb {R}}^{D}$ for the candidate pair (*i*, *j*). The two representations are concatenated as ${\mathbf{s}}_{ij}=[{\mathbf{e}}_{i}\Vert {\mathbf{e}}_{j}]\in {\mathbb {R}}^{2D}$ and fed into a parameterized nonlinear predictor $\phi _{r^{*}}(\cdot )$:

20 $$\begin{aligned} p_{ij} = \sigma \left( \phi _{r^{*}}({\mathbf{s}}_{ij}) \right) , \end{aligned}$$

where $p_{ij}\in [0,1]$ denotes the confidence that $r^{*}$ holds for (*i*, *j*).

For the training set ${\mathcal {S}}=\{(i,j,y_{ij})\}$, where $y_{ij}\in \{0,1\}$, the model is optimized using binary cross-entropy:

21 $$\begin{aligned} {\mathcal {L}} = - \frac{1}{|{\mathcal {S}}|} \sum _{(i,j,y_{ij})\in {\mathcal {S}}} \left[ y_{ij}\log p_{ij} + (1-y_{ij})\log (1-p_{ij}) \right] , \end{aligned}$$

where $|{\mathcal {S}}|$ denotes the number of training samples and ${\mathcal {L}}$ denotes the overall training objective.

NEOGRAN also maintains a native path-tracing mechanism in the structural branch to generate $\pi _{ij}^{*}$. For each candidate pair, a structural context subgraph ${\mathcal {G}}_{ij}^{(L)}=({\mathcal {V}}_{ij}^{(L)},{\mathcal {E}}_{ij}^{(L)})$ is constructed from the *L*-hop message-passing neighborhoods of *i* and *j* and can be pruned by random-walk-based structural context sampling. For evidence-path extraction, ${\mathcal {G}}^{(L)}_{ij}$ is constructed only from the message-passing graph, so target test edges are not used for path extraction.

During structural propagation, each node $v\in {\mathcal {V}}_{ij}^{(L)}$ maintains a path-tracing score $q_{v}^{(l)}$, a predecessor pointer $\operatorname {pred}_{v}^{(l)}$, and a relation label $\operatorname {rel}_{v}^{(l)}$. Here, $q_{v}^{(l)}$ denotes the best tracing score from the starting disease node *i* to node *v* after layer *l*, while $\operatorname {pred}_{v}^{(l)}$ and $\operatorname {rel}_{v}^{(l)}$ record the predecessor node and relation type. At initialization, $q_{i}^{(0)}=0$ and $q_{v}^{(0)}=-\infty$ for *v≠ i*.

For each transition edge $u\xrightarrow {r}v$ at layer *l*, NEOGRAN evaluates the consistency between ${\mathbf{W}}_{r}^{(l)}{\mathbf{h}}_{u}^{(l)}$ and ${\mathbf{h}}_{v}^{(l+1)}$. The tracing score is updated as

22 $$\begin{aligned} q_{v}^{(l+1)} = \max \left( q_{v}^{(l)}, \max _{r\in {\mathcal {R}}} \max _{u:(u,r,v)\in {\mathcal {E}}_{ij}^{(L)}} \left[ q_{u}^{(l)} + \operatorname {sim} \left( {\mathbf{W}}_{r}^{(l)}{\mathbf{h}}_{u}^{(l)}, {\mathbf{h}}_{v}^{(l+1)} \right) \right] \right) , \end{aligned}$$

where $\operatorname {sim}(\cdot ,\cdot )$ denotes cosine similarity. The retained term $q_{v}^{(l)}$ allows the evidence path to terminate within at most *L* hops. When the maximum is achieved by $u\xrightarrow {r}v$, $\operatorname {pred}_{v}^{(l+1)}$ and $\operatorname {rel}_{v}^{(l+1)}$ are updated with *u* and *r*. When it is achieved by $q_{v}^{(l)}$, the predecessor pointer and relation label remain unchanged.

After *L* propagation layers, if the target protein node *j* is reachable in ${\mathcal {G}}_{ij}^{(L)}$, NEOGRAN backtracks from *j* to *i* using the recorded pointers and relation labels:

23 $$\begin{aligned} \pi _{ij}^{*} = \left( n_{0}=i \xrightarrow {r_{1}} n_{1} \xrightarrow {r_{2}} \cdots \xrightarrow {r_{K}} n_{K}=j \right) , \quad K\le L, \end{aligned}$$

where $n_{0},n_{1},\ldots ,n_{K}$ denote the nodes on the path, and $r_{1},r_{2},\ldots ,r_{K}$ denote the corresponding relation types. If no valid path exists within depth *L*, the model still outputs $p_{ij}$ but does not return a structural evidence path. Thus, NEOGRAN jointly produces a relation score and a traceable structural evidence path for validation and structural evidence analysis.

## Results

### Implementation

#### Benchmark

The main experiments are conducted on PrimeKG, a large-scale biomedical knowledge graph that integrates 20 biomedical databases and covers 10 entity types. It is therefore suitable for evaluating relation prediction models that use both heterogeneous graph structures and node-associated texts.

As shown in Table 2, PrimeKG contains 129,375 entity nodes and 4,050,249 relational edges, including 17,080 disease nodes, 27,671 gene/protein nodes, and 84,624 nodes of other types. Among all edges, 80,411 are disease–gene/protein edges related to the target task, and the remaining 3,969,838 edges belong to other relation types. PrimeKG is used as the main benchmark for comparative experiments, ablation studies, and evidence-path analyses.

**Table 2:** Node and edge statistics for PrimeKG and BioKG

Knowledge graph	Type of node	Count	Type of edge	Count
PrimeKG	Disease	17080	Disease–gene/protein	80411
	Gene/protein	27671	All other edge types	3969838
	All other node types	84624		
	Total	129375	Total	4050249
BioKG	Disease	5814	Protein–disease	108995
	Gene/protein	59664	All other edge types	1965351
	All other node types	40859		
	Total	106337	Total	2074346

BioKG is used as an independent external validation knowledge graph. As shown in Table 2, BioKG contains 106,337 entity nodes and 2,074,346 relational edges, including 5,814 disease nodes, 59,664 gene/protein nodes, and 40,859 nodes of other types. Among all edges, 108,995 are protein–disease edges related to the validation task, and the remaining 1,965,351 edges are retained as background structural context. The PROTEIN_DISEASE_ASSOCIATION relation is mapped to disease–protein candidate pairs. Because BioKG differs from PrimeKG in graph scale, data sources, relation composition, and local topology, it is used for method-level external validation under retraining and evaluation on an independent knowledge graph.

#### Experimental setup

NEOGRAN is implemented in PyTorch 2.0.1 and trained on an NVIDIA A100 GPU. The structural branch, textual branch, and relation prediction module are jointly optimized. The learning rate is $5\times 10^{-4}$, the weight decay is $1\times 10^{-5}$, the maximum number of epochs is 200, and the early stopping patience is 20. Unless otherwise specified, the default configuration uses a message-positive ratio of 0.70, 3 R-GCN layers, a structural hidden dimension of 256, a fusion width of 768, and 8 attention heads.

The main PrimeKG experiments adopt a held-out target-edge evaluation protocol. For each random split, known disease–protein target edges are divided into training and test portions with an 85:15 ratio. Test target edges are held out from structural message passing, supervised optimization, model selection, negative sampling, and evidence-path extraction. All non-target relation edges are retained as background context, while target disease–protein labels are used only according to the training, validation, and test split. Within the training portion, 70% of target disease–protein edges are retained in the message graph for structural propagation, while the remaining target edges are used only as supervised positive samples. This separates structural propagation from target-relation supervision, so each target edge is assigned to a single role within the experimental split. Model selection is performed only within the PrimeKG training portion through ten-fold cross-validation. Negative samples are generated independently within each split by replacing one endpoint of a positive disease–protein pair under node-type constraints, while known true disease–protein target edges are excluded.

For external validation, BioKG [42] is used after the default model configuration is selected on PrimeKG. The same split principle is applied by separating target validation edges from the message graph. BioKG is not used for PrimeKG training, validation, or hyperparameter selection.

#### Baseline methods

We compare NEOGRAN with TxGNN, FuseLinker, BioMedKG, BioGraphFusion, and KDGene, which represent representative biomedical graph neural network, text-enhanced graph modeling, multimodal joint representation learning, graph–semantic collaborative reasoning, and disease–gene prediction-oriented biomedical knowledge graph modeling, respectively. TxGNN provides a strong biomedical GNN baseline, FuseLinker introduces large language model-derived text embeddings into GNN-based link prediction, BioMedKG combines language model representations, graph contrastive learning, and knowledge graph embeddings, BioGraphFusion coordinates global semantic modeling with graph structure propagation, and KDGene provides a disease–gene prediction-oriented biomedical knowledge graph baseline. All baselines were trained and evaluated using the same data splits and negative sampling constraints as NEOGRAN, while following their originally supported module settings. Therefore, the baseline comparison assesses end-to-end method-level performance, whereas the specific contributions of individual components are further elucidated through ablation studies.

#### Evaluation metrics

This study uses AUPR, AUROC, F1-score, and MCC as the main evaluation metrics under 1:1 and 1:3 positive-to-negative sampling ratios. Let $\textrm{TP}$, $\textrm{FP}$, $\textrm{TN}$, and $\textrm{FN}$ denote true positives, false positives, true negatives, and false negatives, respectively. F1-score and MCC are defined as

24 $$\begin{aligned} \textrm{F1}=\frac{2\times \textrm{TP}}{2\times \textrm{TP}+\textrm{FP}+\textrm{FN}}, \end{aligned}$$

25 $$\begin{aligned} \textrm{MCC}= \frac{\textrm{TP}\times \textrm{TN}-\textrm{FP}\times \textrm{FN}}{\sqrt{(\textrm{TP}+\textrm{FP})(\textrm{TP}+\textrm{FN})(\textrm{TN}+\textrm{FP})(\textrm{TN}+\mathrm {FN)}}}. \end{aligned}$$

AUPR and AUROC evaluate ranking and discriminative ability across thresholds:

26 $$\begin{aligned} \textrm{AUPR}=\int _{0}^{1}\textrm{Precision}(\textrm{Recall})\,d\textrm{Recall}, \end{aligned}$$

27 $$\begin{aligned} \textrm{AUROC}=\int _{0}^{1}\textrm{TPR}(\textrm{FPR})\,d\textrm{FPR}, \end{aligned}$$

where $\textrm{TPR}$ is equivalent to Recall and $\textrm{FPR}$ is defined as

28 $$\begin{aligned} \textrm{TPR}=\frac{\textrm{TP}}{\textrm{TP}+\textrm{FN}}, \qquad \textrm{FPR}=\frac{\textrm{FP}}{\textrm{FP}+\textrm{TN}}. \end{aligned}$$

Because disease–protein relation prediction is usually imbalanced, AUPR emphasizes positive-relation identification, while AUROC, F1-score, and MCC measure threshold-dependent or threshold-independent discriminative performance from complementary perspectives.

For the filtered all-candidate ranking experiment, we further report MRR, Recall@K, and NDCG@K. For each test disease, all gene/protein nodes are treated as candidate proteins after filtering known disease–protein target edges used for training, message passing, or model selection. The held-out test proteins associated with the disease are retained as relevant items. MRR measures the reciprocal rank of the first held-out positive protein averaged over test diseases, Recall@K measures the fraction of held-out positive proteins recovered among the top K candidates, and NDCG@K evaluates the position-weighted ranking quality within the top K list. These ranking metrics are used to evaluate candidate prioritization performance in the all-candidate setting.

### Empirical performance analysis

This section evaluates NEOGRAN through hyperparameter sensitivity analysis and benchmark comparison experiments on PrimeKG, followed by external validation on BioKG.

#### Main benchmark results on PrimeKG

We first analyze the sensitivity of key hyperparameters on PrimeKG. Detailed heatmaps are provided in Supplementary Fig. S1, where cross-validation AUPR is evaluated under different combinations of the message-positive ratio, the number of R-GCN layers, the R-GCN hidden dimension, the fusion width, and the number of attention heads. The best or near-best performance appears in the medium-capacity range, suggesting a balance between structural receptive field, representation capacity, and graph–text interaction. Based on this analysis, the default configuration is set to a message-positive ratio of 0.70, 3 R-GCN layers, a structural hidden dimension of 256, a fusion width of 768, and 8 attention heads.

**Fig. 2:**
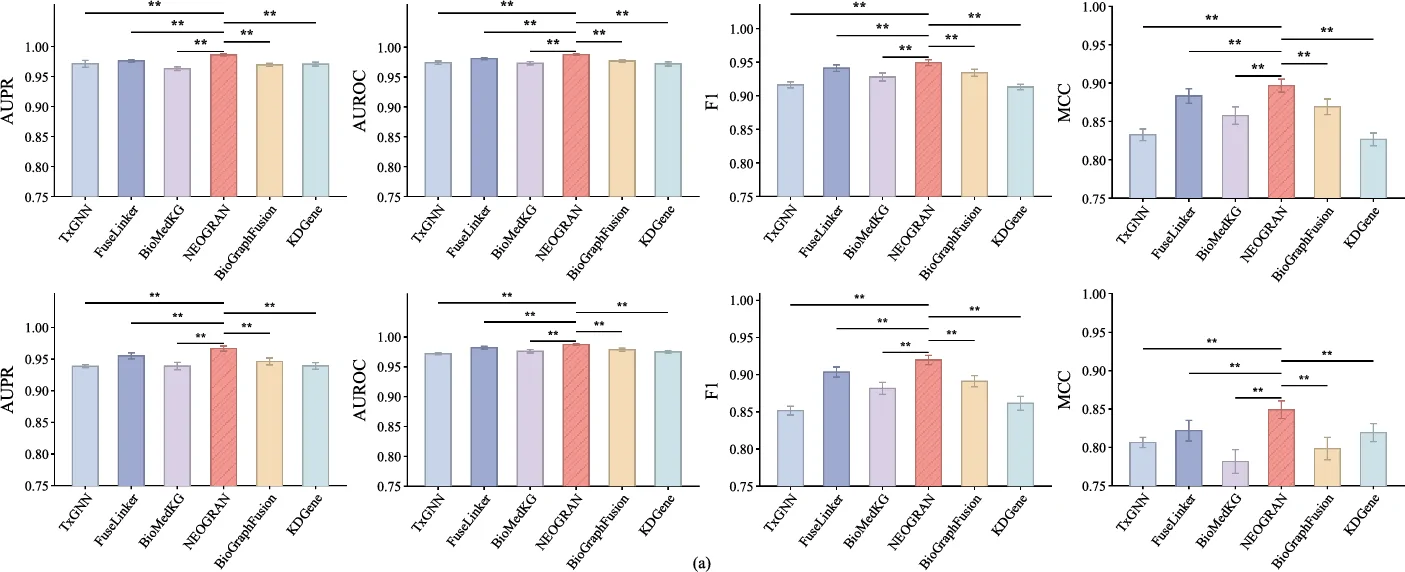
Performance comparison between NEOGRAN and representative baseline methods. The bar plots show the average performance of TxGNN, FuseLinker, BioMedKG, NEOGRAN, BioGraphFusion, and KDGene across four sampled-classification metrics: AUPR, AUROC, F1, and MCC. Error bars indicate the standard deviations over 10 independent outer splits. Significance was assessed using a two-sided paired Student’s *t*-test across the 10 independent outer splits, where ^**^ denotes *P<0.01*. **a** A 1:1 positive-to-negative sampling ratio. **b** A 1:3 positive-to-negative sampling ratio

To evaluate relation prediction performance, NEOGRAN is compared with TxGNN, FuseLinker, BioMedKG, BioGraphFusion, and KDGene on PrimeKG. All methods use the same outer random splits and type-constrained negative sampling strategy. Fig 2 reports the results under 1:1 and 1:3 positive-to-negative sampling ratios. These sampled settings are used as controlled binary classification benchmarks to evaluate discriminative ability under balanced and moderately imbalanced positive–negative ratios. The bars show the mean performance over 10 independent outer splits, and the error bars indicate the standard deviation. The complete numerical values corresponding to Fig. 2 are provided in Supplementary Table S1.

Under the 1:1 setting, NEOGRAN achieves the best performance across all four reported sampled-classification metrics, with an AUPR of 0.9860 and an AUROC of 0.9875. This indicates reliable disease–protein association prediction under a balanced sampled setting. The advantage across threshold-independent and threshold-based sampled classification metrics reflects the joint contribution of relation-aware structural modeling, entity-level textual identifier encoding, and bidirectional structural–textual interaction.

Under the 1:3 setting, NEOGRAN still achieves the best overall performance across the four reported metrics, with an AUPR of 0.9668 and an MCC of 0.8491. The sustained AUPR advantage shows effectiveness when negative candidates become more dominant in the sampled evaluation setting, while the high MCC indicates balanced classification under a larger negative sampling ratio. These results suggest that the performance gain is consistent with the benefit of structural–textual collaboration under controlled sampled classification.

To further evaluate whether the models can prioritize disease–protein associations in a more discovery-oriented setting, we conducted a filtered all-candidate ranking experiment on PrimeKG. For each test disease, all gene/protein nodes were used as candidate proteins, while known disease–protein target edges used for training, message passing, or model selection were filtered out. Each model then scored all remaining disease–protein candidate pairs, and the held-out test proteins were ranked among the full candidate set.

**Fig. 3:**
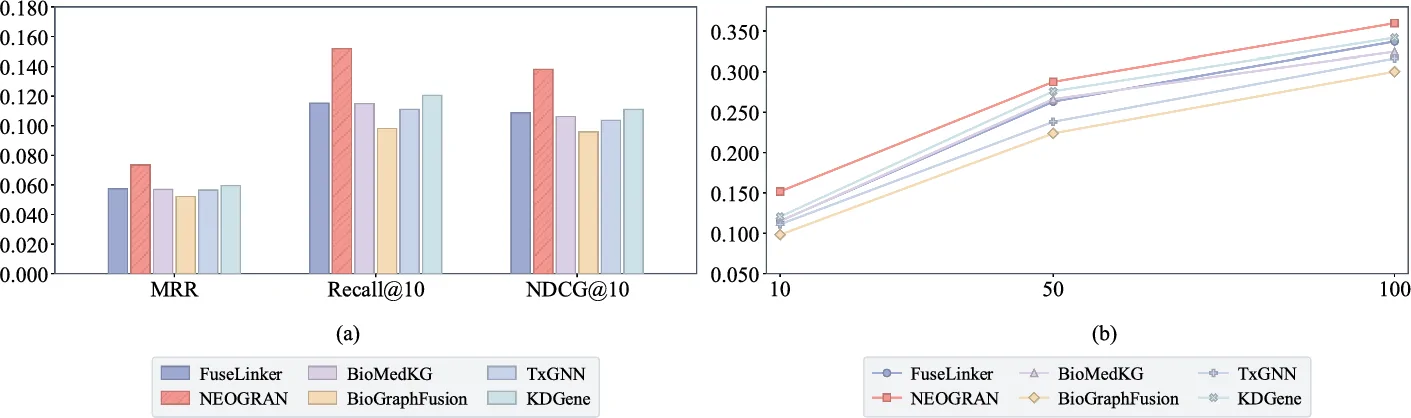
Filtered all-candidate ranking evaluation on PrimeKG. For each test disease, all gene/protein nodes were used as candidate proteins after filtering known disease–protein target edges used for training, message passing, or model selection. **a** Early-ranking performance measured by MRR, Recall@10, and NDCG@10. **b** Recall@K at K = 10, 50, and 100. NEOGRAN shows the strongest performance in both early-ranking metrics and Recall@K across K = 10, 50, and 100

As shown in Fig. 3, NEOGRAN shows consistently strong all-candidate ranking performance. NEOGRAN obtains the highest MRR among all compared methods, indicating stronger early retrieval ability in the filtered all-candidate setting. NEOGRAN also achieves the highest Recall@10 and NDCG@10, indicating that it is more effective in placing high-confidence disease–protein candidates at the top of the ranked list. At larger cutoff values, NEOGRAN continues to achieve the highest Recall@50 and Recall@100, suggesting stronger Top-K candidate coverage in the filtered all-candidate setting. These results indicate that NEOGRAN improves both early high-confidence candidate prioritization and broader Top-K candidate retrieval on PrimeKG. The complete numerical values corresponding to Fig. 3 are provided in Supplementary Table S3.

We further compared NEOGRAN with representative KGE-based models and BERT-based text encoder variants as an auxiliary analysis. NEOGRAN achieved the best overall performance among the tested configurations, supporting the effectiveness of its graph–text collaborative design. Detailed AUPR-related curve results and numerical values are provided in Supplementary Fig. S2.

Although NEOGRAN shows strong sampled-classification performance and favorable early-ranking ability on PrimeKG, results from a single knowledge graph may be influenced by specific entity coverage, relation distribution, and local topology. Therefore, external validation on BioKG is conducted to examine method-level robustness under retraining and evaluation on an independent knowledge graph.

#### External validation on BioKG

For method-level external validation on an independent knowledge graph, NEOGRAN is retrained and evaluated on the BioKG dataset. Disease–protein relation prediction remains the target task, and the same target-edge separation strategy, type-constrained negative sampling strategy, and evaluation metrics are used. For a fair comparison, NEOGRAN and all baselines are retrained and evaluated under the same BioKG splits and negative sampling settings.

**Fig. 4:**
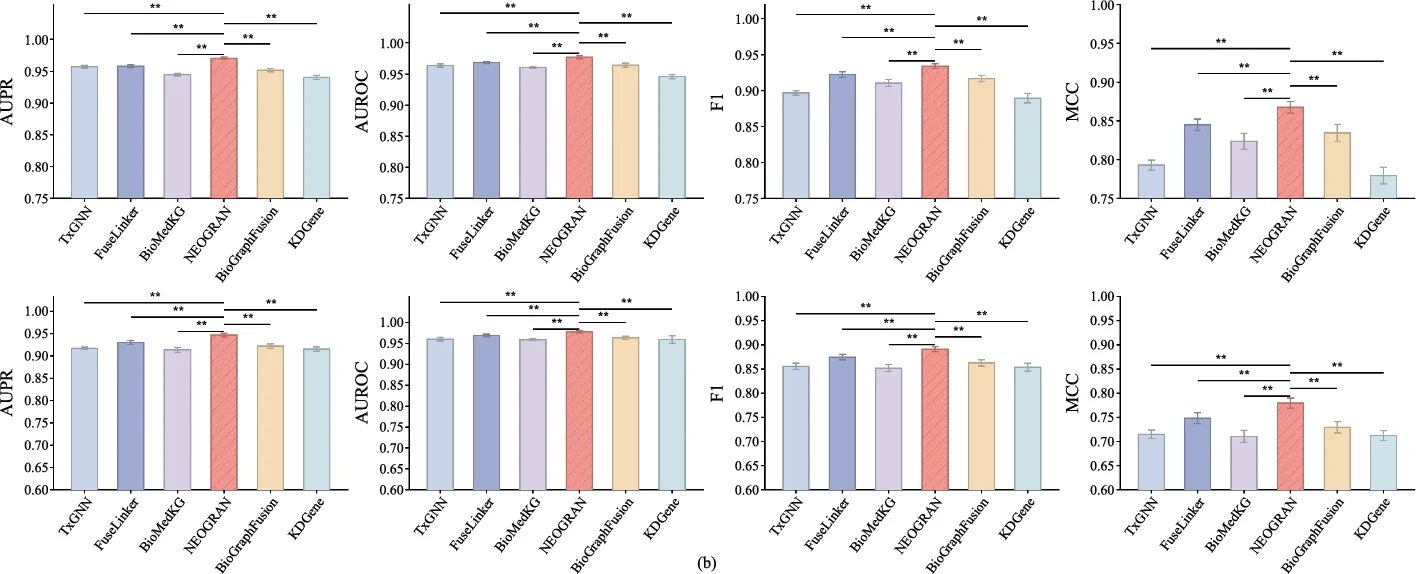
External validation results on BioKG. The bar plots show the performance of TxGNN, FuseLinker, BioMedKG, NEOGRAN, BioGraphFusion, and KDGene on the independent BioKG dataset across four sampled-classification metrics: AUPR, AUROC, F1, and MCC. Error bars indicate the standard deviations over 10 independent outer splits. Significance was assessed using a two-sided paired Student’s *t*-test across the 10 independent outer splits, where ^**^ denotes *P<0.01*. **a** A 1:1 positive-to-negative sampling ratio. **b** A 1:3 positive-to-negative sampling ratio

Figure 4 reports the external validation results on BioKG. The complete numerical values corresponding to Fig. 4 are provided in Supplementary Table S2. Across both sampling ratios, NEOGRAN achieves the best overall performance. In the 1:1 setting, NEOGRAN achieves the best performance across all four reported sampled-classification metrics, with an AUPR of 0.9706 and an AUROC of 0.9774. This indicates that NEOGRAN remains effective under balanced sampled classification on an independent biomedical knowledge graph. The consistent advantage across AUPR, AUROC, F1, and MCC supports the method-level robustness of the structural–textual collaborative framework under external retraining and evaluation. In the 1:3 setting, NEOGRAN still achieves the best overall performance across the four reported metrics. The sustained advantage under a larger negative sampling ratio suggests that NEOGRAN remains robust when negative candidates become more dominant in the sampled evaluation setting.

### Ablation analysis

To evaluate the contribution of the three core modules, namely the structural branch, the textual branch, and the bidirectional cross-attention module, we conduct ablation experiments on PrimeKG under the same data splits, target-edge separation strategy, and negative sampling strategy as the main experiments. Table 3 compares the Structural + Textual configuration of NEOGRAN with variants that remove one modality branch or replace the bidirectional cross-attention module with weaker fusion mechanisms. Specifically, Textual-only removes the structural branch, Structural-only removes the textual branch, Avg fusion and Concat fusion replace bidirectional cross-attention with shallow fusion, and the two one-way attention variants retain only one direction of cross-modal conditioning. The Structural + Textual configuration achieves the best performance across all reported metrics, indicating that its advantage comes from the coordinated use of structural information, name/type-based textual cues, and bidirectional structural–textual interaction.

**Table 3:** Ablation results for the structural branch, textual branch, and bidirectional cross-attention module of NEOGRAN

Variant	AUPR	AUROC	F1	MCC	*Δ * AUPR
**Structural + Textual**	**0**.**9860** **± 0.0017**	**0**.**9875** **± 0.0015**	**0**.**9492** **± 0.0043**	**0**.**8967** **± 0.0085**	–
Textual-only	0.9778 ± 0.0024	0.9802 ± 0.0021	0.9364 ± 0.0052	0.8711 ± 0.0098	−0.0082
Structural-only	0.9794 ± 0.0022	0.9820 ± 0.0019	0.9395 ± 0.0049	0.8768 ± 0.0093	−0.0066
Avg fusion	0.9736 ± 0.0028	0.9788 ± 0.0024	0.9328 ± 0.0055	0.8665 ± 0.0105	−0.0124
Concat fusion	0.9751 ± 0.0026	0.9797 ± 0.0023	0.9351 ± 0.0052	0.8704 ± 0.0101	−0.0109
S*→ *T attention	0.9821 ± 0.0021	0.9846 ± 0.0018	0.9436 ± 0.0048	0.8855 ± 0.0092	−0.0039
T*→ *S attention	0.9829 ± 0.0020	0.9850 ± 0.0018	0.9444 ± 0.0046	0.8870 ± 0.0090	−0.0031

Structural + Textual denotes the complete NEOGRAN configuration with both structural and textual branches and bidirectional cross-attention. Textual-only removes the structural branch and uses only the textual branch; Structural-only removes the textual branch and uses only the structural branch. Avg fusion and Concat fusion replace bidirectional cross-attention with shallow fusion strategies. S denotes structural representation, and T denotes textual representation. Boldface identifies the best-performing variant, and bold values indicate the best results.

For the structural and textual branches, both single-modality variants perform worse than the Structural + Textual configuration. The Textual-only variant removes the structural branch and decreases AUPR from 0.9860 to 0.9778, corresponding to a drop of 0.0082. This indicates that name/type-based textual cues alone cannot fully capture relation constraints in heterogeneous biomedical knowledge graphs. The Structural-only variant removes the textual branch and decreases AUPR to 0.9794, corresponding to a drop of 0.0066. This shows that structural information alone cannot capture the complementary entity-level cues provided by node-associated text. Thus, disease–protein relation prediction benefits from the joint use of structurally derived relational evidence and text-derived entity-level cues.

For the bidirectional cross-attention module, replacing it with Avg fusion and Concat fusion produces larger AUPR drops of 0.0124 and 0.0109, respectively. This indicates that simple late fusion cannot fully exploit the complementarity between structural neighborhoods and name/type-based textual cues. The one-way attention variants reduce the AUPR drops to 0.0039 for S*→ *T attention and 0.0031 for T*→ *S attention, but both remain inferior to the Structural + Textual configuration. These results suggest that the three core modules all contribute to final performance: the structural branch provides relation-aware structural constraints, the textual branch provides entity-level textual cues, and bidirectional cross-attention further improves prediction by enabling mutual conditioning between the two representation spaces.

### Evidence-path analysis

High predictive performance does not necessarily guarantee model verifiability. In disease–protein relation prediction, the key question is whether high-confidence predictions are supported by biomedically meaningful structural evidence. To assess the evidence paths generated by NEOGRAN, we remove model-generated evidence paths and length-matched random paths separately on PrimeKG and compare the resulting drops in prediction scores. A larger drop after evidence-path removal indicates that these paths are not generic connected subgraphs, but structural support associated with the prediction score.

**Fig. 5:**
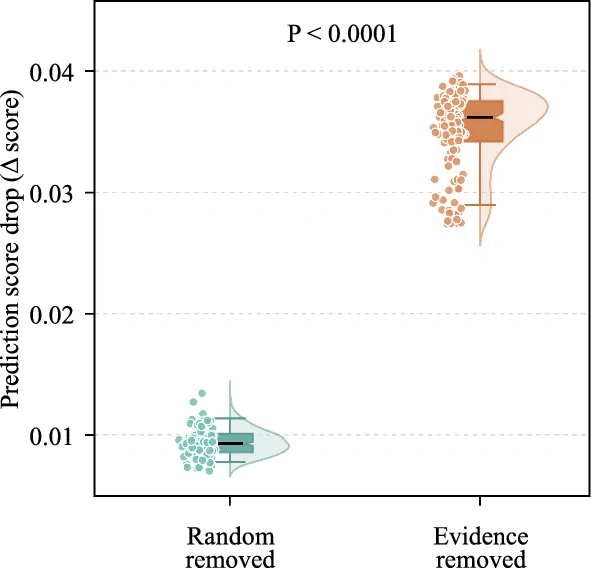
Distribution of prediction-score drops after removing evidence paths and length-matched random paths. The figure shows the score drops for 11,097 predicted disease–protein node pairs. Evidence removed denotes the removal of evidence paths generated by NEOGRAN, whereas Random removed denotes the removal of length-matched random paths. The significance of the difference between the two groups was evaluated using the Wilcoxon signed-rank test

Figure 5 shows the overall results for 11,097 predicted node pairs. Compared with random path deletion, evidence path deletion leads to a significantly larger decrease in prediction scores under the Wilcoxon signed-rank test (*P<0.0001*). Random path deletion causes only minor perturbations, suggesting that high scores do not arise from general graph connectivity. In contrast, the clear degradation after evidence path deletion indicates that NEOGRAN captures structural context associated with candidate disease–protein relations, providing perturbation-based evidence that these paths are linked to model outputs.

**Fig. 6:**
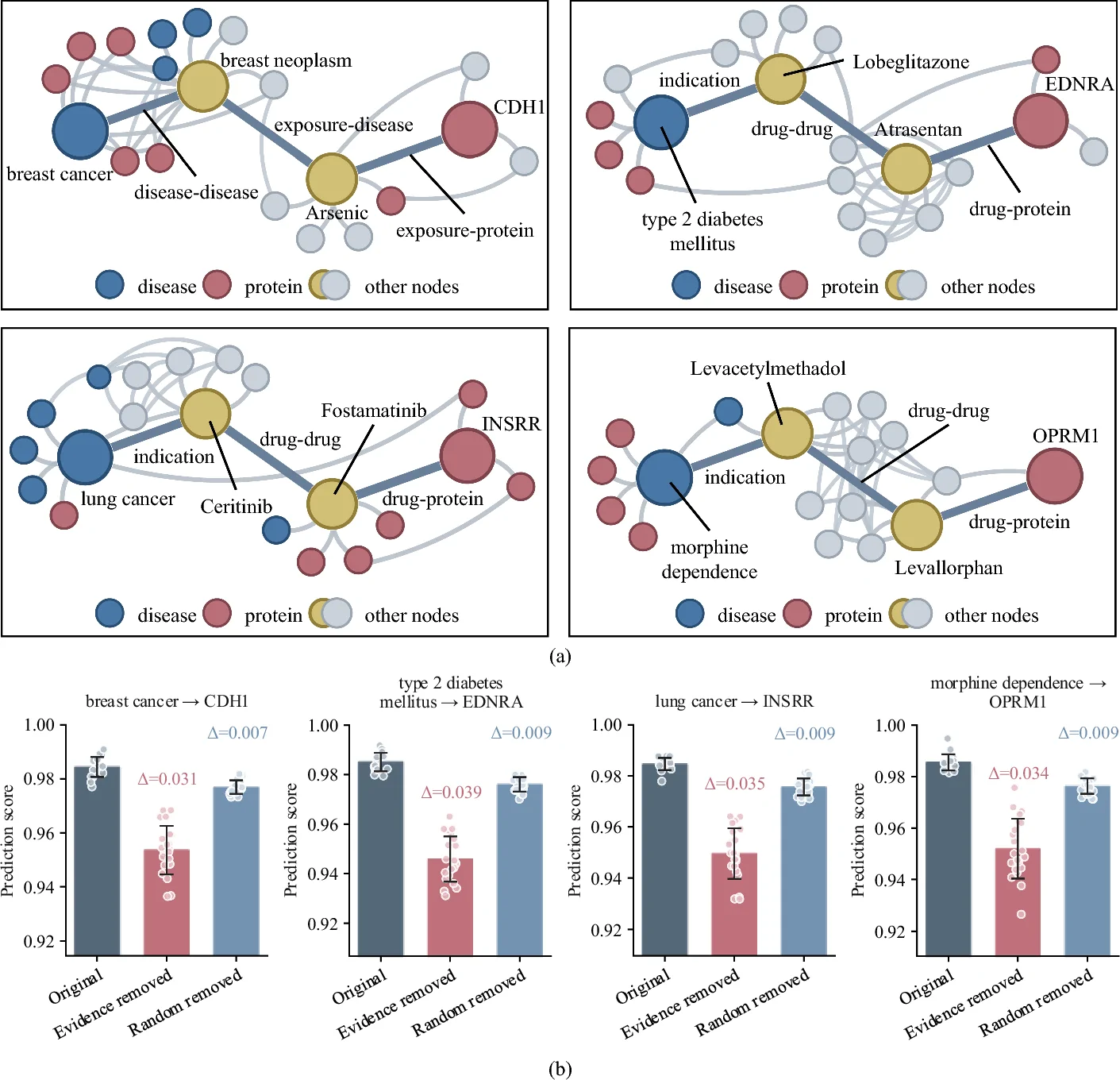
Representative evidence paths generated by NEOGRAN and the corresponding case-level path-removal results. The upper panel shows traceable evidence paths for four disease–protein prediction cases. Dark edges denote key structural evidence extracted by the model, while light-colored nodes and edges denote the related neighborhood context. The lower panel shows changes in prediction scores under the original setting, after removing the evidence paths, and after removing length-matched random paths. Original denotes the original prediction score, Evidence removed denotes the prediction score after removing the model-generated evidence paths, and Random removed denotes the prediction score after removing length-matched random paths

Figure 6 further illustrates four representative cases. For breast cancer (*→ *) CDH1, type 2 diabetes mellitus (*→ *) EDNRA, lung cancer (*→ *) INSRR, and morphine dependence (*→ *) OPRM1, evidence-path removal results in larger score drops than random-path removal. For example, type 2 diabetes mellitus (*→ *) EDNRA shows a drop of 0.039 after evidence-path removal but only 0.009 after random-path removal, and morphine dependence (*→ *) OPRM1 shows corresponding drops of 0.034 and 0.009. These cases indicate that the model-generated paths show a stronger association with local prediction scores than arbitrary neighborhood links.

From a biomedical perspective, some evidence paths are consistent with prior findings. For type 2 diabetes mellitus (*→ *) EDNRA, the model path passes through lobeglitazone and atrasentan, linking the diabetes treatment context to EDNRA-related targets; previous studies have shown that atrasentan is clinically relevant to renal events in patients with type 2 diabetes and chronic kidney disease [43]. For morphine dependence (*→ *) OPRM1, large-scale studies have supported the genetic association between OPRM1 and opioid use disorder [44]. For breast cancer (*→ *) CDH1 and lung cancer (*→ *) INSRR, the paths should be viewed as candidate evidence identified within the current graph structure and semantic context. They do not provide direct causal explanations, but offer prioritized leads for literature review, hypothesis generation, and further biological validation.

Overall, the evidence-path analysis shows that NEOGRAN does not merely present paths as post-hoc explanations. Instead, its explanatory ability is supported by structural evidence associated with prediction scores. The path-removal experiment provides perturbation-based evidence that the generated paths are linked to model outputs, and representative cases show their potential consistency with biomedical knowledge. These findings suggest that the generated paths can provide useful structural evidence for hypothesis generation. Thus, NEOGRAN improves disease–protein relation prediction while providing traceable and testable structural support for candidate relations.

## Discussion

NEOGRAN’s methodological contribution lies in its three-layer relation-aware graph neural network architecture and its task-specific coupling of structural encoding, textual encoding, bidirectional cross-attention, gated fusion, and evidence-path tracing for disease–protein relation prediction in biomedical knowledge graphs. Unlike methods that use textual embeddings as static node features or combine graph and text representations through concatenation, averaging, score-level fusion, or one-way attention, NEOGRAN uses bidirectional cross-attention to enable reciprocal conditioning between structural and textual representations. Specifically, the Structural-to-Textual direction refines structural node representations with textual cues, whereas the Textual-to-Structural direction constrains textual representations with sampled local structural memory. This complementary coupling allows graph-derived relational information and textual representations to interact within the same candidate-pair scoring process. The ablation results further show that the structural branch, textual branch, and bidirectional cross-attention module contribute complementary gains, indicating that NEOGRAN’s performance improvement comes from their coordinated design rather than from any single component alone. The filtered all-candidate ranking results further show that NEOGRAN improves both early candidate prioritization, as reflected by MRR, Recall@10, and NDCG@10, and broader Top-*K* candidate coverage, as reflected by Recall@50 and Recall@100.

The evidence-path analysis further highlights the methodological value that distinguishes NEOGRAN from general black-box prediction models. In biomedical settings, a high prediction score alone is insufficient to support the credibility of a candidate disease–protein relation. Researchers also need to examine which relational chains are associated with the model output. The evidence paths generated by NEOGRAN constitute prediction-associated structural evidence derived from structural propagation and relation scoring. The path-removal experiments show that removing model-generated evidence paths causes a larger decrease in prediction scores than removing length-matched random paths. This indicates that these paths provide structural support directly associated with model outputs. Accordingly, NEOGRAN can transform relation prediction results into candidate evidence that is easier to examine, review, and further validate. It also provides a clearer starting point for hypothesis generation, literature-based evidence review, candidate prioritization, and further biological validation.

Several limitations remain in this study. First, the predictive performance and evidence quality of NEOGRAN are still affected by the coverage of knowledge graphs, the quality of node-associated texts, and the completeness of supervised relation labels. Missing relations, bias toward frequently studied entities, and ambiguous entity names may all influence model outputs. Second, the current evidence paths mainly reflect structural support within the knowledge graph. They are not equivalent to complete causal mechanistic explanations and therefore still require validation using literature evidence, experimental findings, and domain expert judgment. Third, although external validation on BioKG supports method-level generalizability across knowledge graph sources, it remains a retrospective computational validation. It does not directly demonstrate the effectiveness of the model in cross-institutional data environments or prospective experimental settings. Future work may further integrate retrieval-augmented reasoning, literature-level evidence snippets, and semantic consistency checking to extend structural paths into more complete verifiable evidence chains. It may also explore zero-shot or few-shot prediction for new diseases, new proteins, and emerging relation types, thereby improving the applicability of NEOGRAN in open, sparse, and continuously updated biomedical knowledge discovery scenarios.

## Conclusions

This study addresses the translationally relevant problem of disease–protein relation prediction in biomedical knowledge graphs and proposes NEOGRAN, a graph–text fusion framework with traceable evidence support. Disease–protein relations connect disease phenotypes, molecular mechanisms, and potential intervention targets. Therefore, their prediction requires not only strong discriminative performance but also results that can be understood, examined, and further validated by researchers. To address the insufficient use of structural information and textual representations, shallow graph–text fusion, and lack of process-level evidence support in existing methods, NEOGRAN integrates relation-aware structural encoding, entity-level textual representation learning, bidirectional cross-modal attention, and native evidence-path tracing into a unified prediction process. Unlike models that focus only on edge prediction performance, the core contribution of NEOGRAN is to unify prediction accuracy, structural–textual complementarity, and evidence traceability, thereby providing a stronger methodological basis for prioritizing candidate disease–protein relations.

The held-out target-edge evaluation on PrimeKG and the external validation on BioKG show that NEOGRAN achieves strong sampled-classification performance and favorable early-rank prioritization compared with representative graph-based, text-enhanced, and multimodal baseline methods. Ablation experiments and path-removal experiments further support the contribution of graph–text interaction and evidence paths to the prediction results. These findings indicate that NEOGRAN is not merely applicable to the local topological patterns of a specific knowledge graph, but provides a generalizable biomedical informatics modeling strategy. Its potential application lies in providing interpretable prioritization of candidate disease–protein relations for hypothesis generation, literature-based evidence review, and further biological validation. Therefore, NEOGRAN can serve as an evidence-supported computational tool in translational bioinformatics for identifying disease–protein associations that merit further validation in complex, incomplete, and continuously evolving biomedical knowledge spaces.

## Additional file

Additional file 1. Supplementary figures and tables. Description: This file provides supplementary experimental results for NEOGRAN, including hyperparameter sensitivity analysis, auxiliary model comparisons, complete numerical performance tables, and filtered all-candidate ranking results.

## Data Availability

The original datasets used in this study are publicly available. PrimeKG is available from Harvard Dataverse at https://doi.org/10.7910/DVN/IXA7BM, and the corresponding resource article is cited as Chandak et al., Building a knowledge graph to enable precision medicine. BioKG is available from the official GitHub repository at https://github.com/dsi-bdi/biokg, with release biokg-v1.0.0 used in this study, and the corresponding resource article is cited as Walsh et al., BioKG: A Knowledge Graph for Relational Learning On Biological Data. Additional experimental results supporting the findings of this study are included in the supplementary material.
