# Supplementary material for: NEOGRAN: traceable graph-text fusion for disease–protein relation prediction in biomedical knowledge graphs
Source: BMC Bioinformatics. 2026 Aug 8;27:267. doi: 10.1186/s12859-026-06580-3 (PMC13746616; doi:10.1186/s12859-026-06580-3)
Supplement: Supplementary file 1 [file 12859_2026_6580_MOESM1_ESM.pdf]

Supplementary Material

NEOGRAN: Traceable Graph-Text Fusion for Disease–Protein Relation Prediction in Biomedical Knowledge Graphs

Supplementary Figure

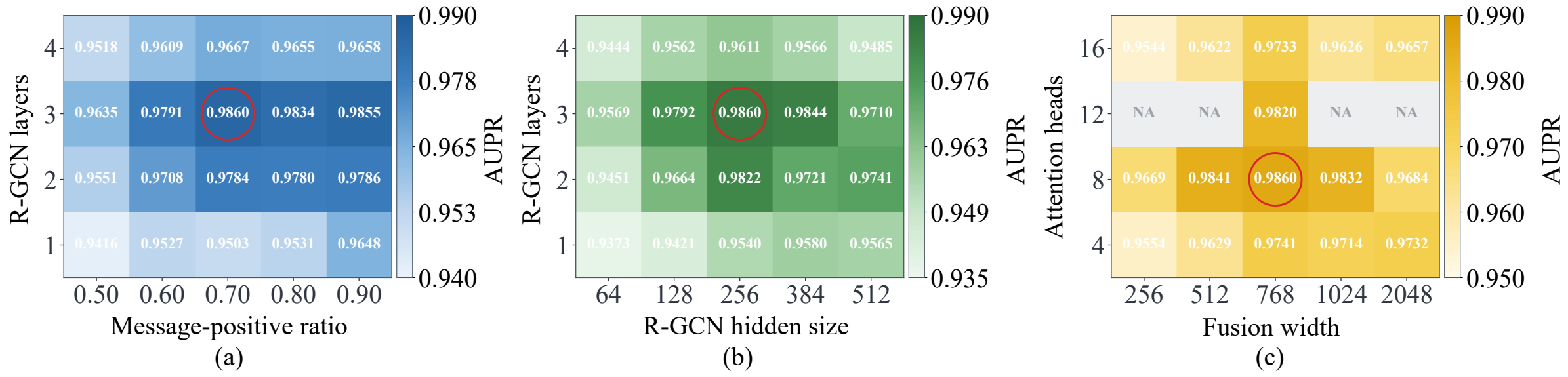

Figure S1: Sensitivity analysis of key hyperparameters in NEOGRAN. The three heatmaps show the variation in cross-validation AUPR under different hyperparameter combinations: (a) message-positive ratio and the number of R-GCN layers; (b) R-GCN hidden dimension and the number of R-GCN layers; (c) number of attention heads and fusion width. Darker colors indicate higher AUPR values.

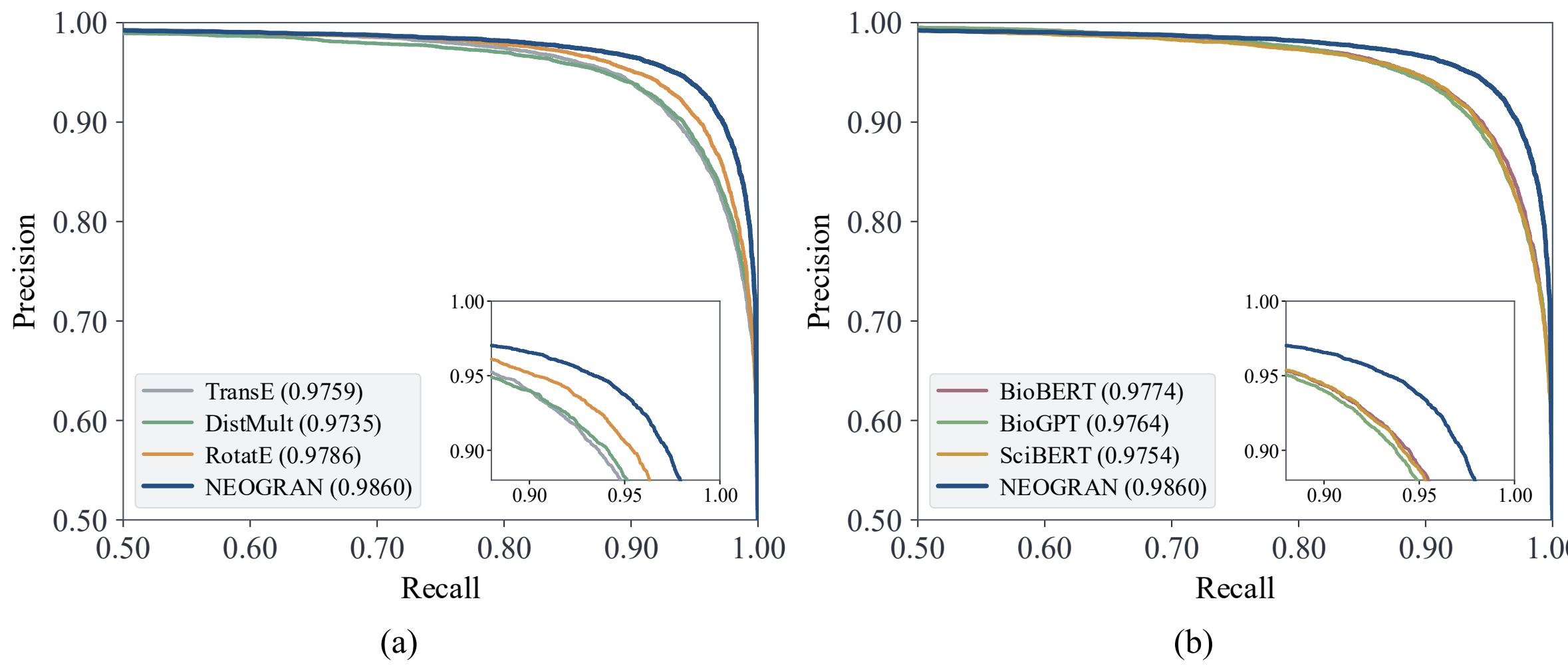

Figure S2: Auxiliary comparison between NEOGRAN and representative KGE-based models and BERT-based text encoder variants. The precision–recall curves show relation prediction performance under different model or encoder configurations, with the values in parentheses indicating the corresponding AUPR. (a) Comparison between NEOGRAN and representative KGE-based models. (b) Comparison between NEOGRAN and BERT-based text encoder variants.

Supplementary Tables

Table S1: Complete numerical results corresponding to Figure 2. Performance comparison between NEOGRAN and representative baseline methods on PrimeKG under 1:1 and 1:3 positive to negative sampling ratios. Values are reported as mean ± standard deviation over 10 independent outer splits.

Sampling ratio	Method	AUPR	AUROC	F1	MCC
1:1	NEOGRAN	**0.9860 ± 0.0017**	**0.9875 ± 0.0015**	**0.9492 ± 0.0043**	**0.8967 ± 0.0085**
1:1	TxGNN	0.9712 ± 0.0057	0.9738 ± 0.0029	0.9161 ± 0.0045	0.8325 ± 0.0076
1:1	FuseLinker	0.9761 ± 0.0022	0.9808 ± 0.0019	0.9411 ± 0.0048	0.8831 ± 0.0094
1:1	BioMedKG	0.9629 ± 0.0031	0.9731 ± 0.0028	0.9279 ± 0.0059	0.8576 ± 0.0113
1:1	BioGraphFusion	0.9694 ± 0.0027	0.9769 ± 0.0024	0.9342 ± 0.0053	0.8690 ± 0.0102
1:1	KDGene	0.9706 ± 0.0033	0.9719 ± 0.0034	0.9130 ± 0.0041	0.8266 ± 0.0083
1:3	NEOGRAN	**0.9668 ± 0.0041**	**0.9875 ± 0.0016**	**0.9198 ± 0.0062**	**0.8491 ± 0.0115**
1:3	TxGNN	0.9389 ± 0.0025	0.9718 ± 0.0015	0.8515 ± 0.0059	0.7823 ± 0.0067
1:3	FuseLinker	0.9552 ± 0.0048	0.9821 ± 0.0023	0.9035 ± 0.0068	0.8218 ± 0.0134
1:3	BioMedKG	0.9391 ± 0.0059	0.9761 ± 0.0030	0.8816 ± 0.0081	0.7816 ± 0.0155
1:3	BioGraphFusion	0.9464 ± 0.0054	0.9786 ± 0.0027	0.8912 ± 0.0075	0.7984 ± 0.0146
1:3	KDGene	0.9393 ± 0.0052	0.9749 ± 0.0023	0.8615 ± 0.0093	0.7912 ± 0.0117

Table S2: Complete numerical results corresponding to Figure 4. External validation performance on BioKG under 1:1 and 1:3 positive to negative sampling ratios. Values are reported as mean ± standard deviation.

Sampling ratio	Method	AUPR	AUROC	F1	MCC
1:1	NEOGRAN	**0.9706 ± 0.0019**	**0.9774 ± 0.0025**	**0.9341 ± 0.0033**	**0.8678 ± 0.0075**
1:1	FuseLinker	0.9582 ± 0.0024	0.9687 ± 0.0017	0.9225 ± 0.0038	0.8450 ± 0.0074
1:1	BioMedKG	0.9449 ± 0.0021	0.9608 ± 0.0018	0.9106 ± 0.0049	0.8237 ± 0.0103
1:1	BioGraphFusion	0.9518 ± 0.0027	0.9646 ± 0.0034	0.9168 ± 0.0043	0.8345 ± 0.0112
1:1	KDGene	0.9406 ± 0.0032	0.9461 ± 0.0036	0.8895 ± 0.0066	0.7793 ± 0.0107
1:1	TxGNN	0.9574 ± 0.0022	0.9640 ± 0.0025	0.8969 ± 0.0031	0.7929 ± 0.0065
1:3	NEOGRAN	**0.9468 ± 0.0043**	**0.9776 ± 0.0026**	**0.8912 ± 0.0052**	**0.7795 ± 0.0105**
1:3	FuseLinker	0.9302 ± 0.0045	0.9690 ± 0.0033	0.8746 ± 0.0058	0.7483 ± 0.0114
1:3	BioMedKG	0.9137 ± 0.0055	0.9586 ± 0.0020	0.8519 ± 0.0071	0.7106 ± 0.0125
1:3	BioGraphFusion	0.9221 ± 0.0050	0.9634 ± 0.0037	0.8627 ± 0.0065	0.7294 ± 0.0116
1:3	KDGene	0.9156 ± 0.0048	0.9591 ± 0.0040	0.8539 ± 0.0083	0.7122 ± 0.0101
1:3	TxGNN	0.9173 ± 0.0028	0.9602 ± 0.0043	0.8556 ± 0.0067	0.7148 ± 0.0088

Table S3: Complete numerical results corresponding to Figure 3. Filtered all-candidate ranking performance on PrimeKG. For each test disease, all gene/protein nodes were considered as candidate proteins after excluding known disease–protein target edges used for training, message passing, or model selection.

Method	MRR	Recall@10	NDCG@10	Recall@50	NDCG@50	Recall@100	NDCG@100
NEOGRAN	**0.0735**	**0.1518**	**0.1381**	**0.2876**	**0.1652**	**0.3598**	**0.1805**
FuseLinker	0.0574	0.1152	0.1088	0.2632	0.1424	0.3372	0.1510
BioMedKG	0.0570	0.1149	0.1062	0.2662	0.1400	0.3246	0.1509
BioGraphFusion	0.0522	0.0981	0.0958	0.2238	0.1165	0.3002	0.1264
TxGNN	0.0565	0.1111	0.1036	0.2381	0.1265	0.3165	0.1474
KDGene	0.0596	0.1205	0.1111	0.2759	0.1489	0.3420	0.1619

Note: MRR = Mean Reciprocal Rank. Recall@K = proportion of true disease–protein associations retrieved within the top-K ranked candidates. NDCG@K = Normalized Discounted Cumulative Gain at rank K, which accounts for both retrieval accuracy and ranking position.
